# Accuracy of RIPASA and Lintula Scores in Diagnosing Acute Appendicitis Using Surgical Findings as the Gold Standard

**DOI:** 10.7759/cureus.31297

**Published:** 2022-11-09

**Authors:** Muhammad Saulat Naeem, Zoya Sadiq, Muhammad Awais, Minhaj Rafi, Sundas Javeed, Itfaque Ahmed, Shahid Farooq, Abrar Ashraf Ali

**Affiliations:** 1 Surgery, Mayo Hospital, Lahore, PAK; 2 Surgery, Naeem Hospital, Gujranwala, PAK; 3 Surgery, Shalamar Medical and Dental College, Lahore, PAK; 4 Plastic Surgery, Services Institute of Medical Sciences, Lahore, PAK

**Keywords:** diagnosing appendicitis, acute appendicitis, lintula, ripasa, surgical findings

## Abstract

Background: Appendicitis is an inflammation of the vermiform appendix's inner lining that spreads to its other sections. Appendectomy is still the standard way to cure appendicitis. The diagnosis of acute appendicitis is still clinical and supported by a raised neutrophilic count and imaging studies; moreover, scoring systems, such as the Raja Isteri Pengiran Anak Saleha Appendicitis (RIPASA) and Lintula scores, help the clinicians in the diagnosis. The main objective of this study was to establish the diagnostic accuracy of RIPASA and Lintula scores for acute appendicitis using surgical findings as the gold standard, in an Asian population.

Methods: This was a retrospective cohort study conducted at the Department of Surgery, Mayo Hospital, Lahore, and Department of Surgery, Services Hospital, Lahore. This study was conducted from January 2020 to January 2022, for the duration of two years. A total of 120 patients were enrolled after meeting the inclusion criteria, and demographic data were obtained. Lintula and RIPASA scores were recorded, and patients were classified as positive or negative based on histopathological findings. IBM SPSS Statistics,* *version 26 (IBM Corp., Armonk, NY) was used to evaluate all of the gathered data.

Results: The average age of the patients in this study was 37.39±14.36 years, with a male-to-female ratio of 1.14:1. Taking surgical finding as the gold standard, RIPASA scoring had a diagnosis accuracy of 91.67% while the Lintula score had a diagnostic accuracy of 79.17%.

Conclusion: While both the RIPASA and the Lintula scoring systems were accurate, the RIPASA scoring system outperformed the Lintula scoring system when surgical findings were used as the gold standard.

## Introduction

Acute inflammation of the appendix is one of the most prevalent surgical emergencies seen in the day-to-day practice around the world [1]. Its prevalence varies between 13% and 77% [2]. In a Pakistan-based research, 36 (48%) of 75 individuals with acute stomach discomfort were identified as acute appendicitis cases [3]. It is still tough to diagnose, especially in children who can show symptoms of a variety of inflammatory diseases similar to acute appendicitis.

Despite its prevalence, making the diagnosis of acute appendicitis is tricky since symptoms and signs of many different gynecological and genitourinary inflammatory processes mimic acute appendicitis; hence, a proper clinical examination is the best way to diagnose acute appendicitis [2,4]. To improve diagnostic accuracy, different scoring systems have been devised. They are affordable, noninvasive, simple to use and duplicate [5-7]. Raja Isteri Pengiran Anak Saleha Appendicitis (RIPASA) scoring is a novel system of diagnostic scoring created for the detection of appendicular inflammation in Asian people [8-10].

The purpose of this study was to see how accurate RIPASA and Lintula scores are at determining acute appendicitis, taking surgical findings as the gold standard. According to the literature, RIPASA and Lintula scoring systems for appendicitis detection have varying degrees of accuracy. However, the literature has a wide range of information. This raises the question of which criteria are more useful and accurate in predicting the severity of acute appendicitis. As a result, we aimed to perform this research to identify current evidence that can help us decide on a more accurate way for diagnosing acute appendicitis, particularly in cases where the diagnosis is negative.

## Materials and methods

This was a retrospective cohort study conducted at the Department of Surgery, Mayo Hospital, Lahore, and Department of Surgery, Services Hospital, Lahore. The duration of this study was of two years, from January 2020 to January 2022. All patients from the ages of 15 to 65 years with pain in the right lower abdomen and symptoms of appendicular inflammation on abdominal examination who underwent surgery for appendicular removal were selected for this study. Patients with incomplete medical records, ruptured appendix (on the medical record) or where Lintula or RIPASA was not mentioned on the records and those with other possible diagnoses like UTI were not included in the study. A total of 120 patients were enrolled after meeting the inclusion criteria.

Medical records of 120 patients were collected from the hospital's medical records department. The initial assessment was done by residents on duty, and RIPASA and Lintula scores were calculated. The patient was classified as positive or negative based on scores. On Lintula, the score was labeled positive if it was ≥21 whereas RIPASA was labeled positive if it was ≥7.5 (Tables 1, 2). These findings were then confirmed by a consultant and recorded in the medical record. After that, histopathological findings were reported as per the consultant pathologist. Histopathology (surgery sample) was labeled as positive if there was presence of inflammation of the appendix, multiple micro-abscesses of the appendiceal wall and severe necrosis of all layers. It was labeled as negative when above-mentioned findings were not noticed. True positive was taken as both a positive score (either Lintula or RIPASA) and positive histopathological findings and vice versa. The accuracy of the results was then calculated after they were compared. All of this data was entered into a spreadsheet.

**Table 1:** Lintula score details FPS, Facial Pain Scale

	Variables	Scores
Gender	Male	2
	Female	0
Intensity of pain (FPS 0-10)	Severe (FPS 7-10)	2
	Mild or moderate (FPS 1-6)	0
Relocation of pain	Yes	4
	No	0
Pain in the right lower abdominal quadrant	Yes	4
	No	0
Vomiting (≥1 episode)	Yes	2
	No	0
Body temperature	≥37.5°C	3
	<37.5°C	0
Guarding	Yes	4
	No	0
Bowel sounds	Absent, tinkling or high-pitched	4
	Normal	0
Rebound tenderness	Yes	7
	No	0

**Table 2:** RIPASA score details RIF, right iliac fossa; NRIC, National Registration Identity Card; WBC, white blood cell

	Variables	Score
Patients	Female	0.5
	Male	1
	Age <39.9 years	1
	Age >40 years	0.5
Symptoms	RIF pain	0.5
	Pain migration to RIF	0.5
	Anorexia	1
	Nausea and vomiting	1
	Duration of symptoms <48 hours	1
	Duration of symptoms >48 hours	0.5
Signs	RIF tenderness	1
	Guarding	2
	Rebound tenderness	1
	Rovsing sign	2
	Fever >37°C <39°C	1
Investigations	Raised WBC count	1
	Negative urine analysis	1
Additional scores	Foreign NRIC	1
Total score		17.5

The sample size of 120 patients was determined with a 95% level of confidence, using the non-probability, consecutive sampling approach.

IBM SPSS Statistics, version 26 (IBM Corp., Armonk, NY) was utilized for data analysis. Means and standard deviations of quantitative factors, such as duration of symptoms, age, RIPASA score, and Lintula score, were computed. The frequency and percentage of qualitative variables like gender and acute appendicitis were also calculated. Using surgical findings as the gold standard, the sensitivity, specificity, positive predictive value (PPV), negative predictive value (NPV), and diagnostic accuracy of the RIPASA and Lintula scores were obtained using 2x2 tables.

## Results

**Figure 1:**
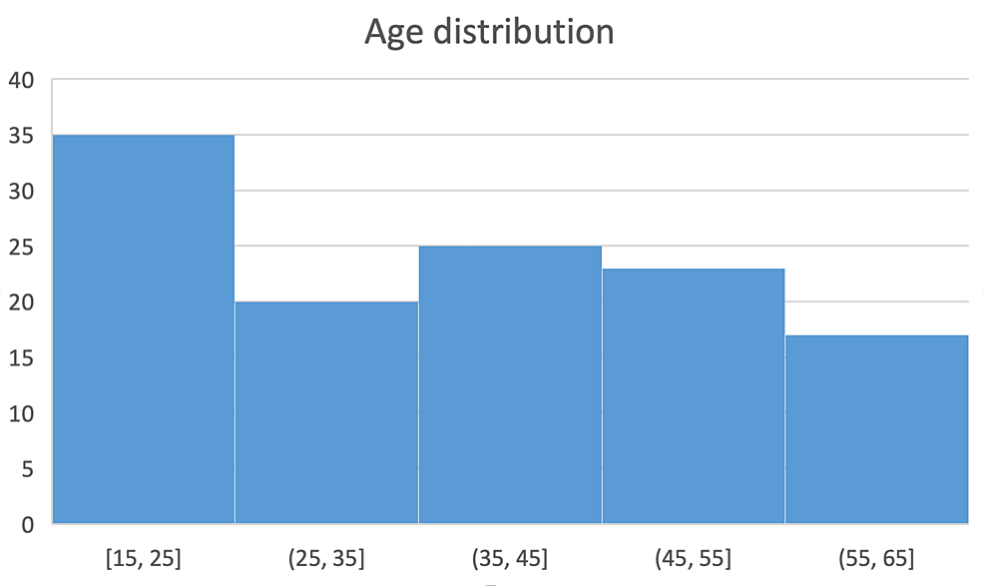
Frequency distribution of age

**Figure 2:**
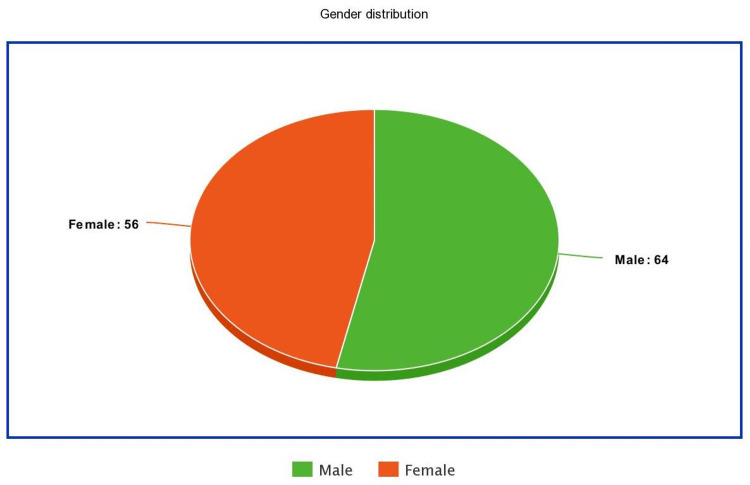
Gender distribution

This research had 120 South Asian patients in total. The patient's average age was 37.39±14.36 years, with the lowest and highest age being 15 and 65 years, respectively (Figure 1). A total of 56 patients (46.67%) were female and 64 patients (53.33%) were male in this research as shown in Figure 2.

The female-to-male ratio among the patients was 1:1.14. According to the findings, the mean number of days a patient spent in pain was 6.62±3.331 days, with the minimum being 1 day and the maximum 12 days (Table 3).

**Table 3:** Study parameters

	Mean	Minimum	Maximum
Age	37.39±14.36 years	15 years	65 years
Duration of patients’ pain	6.62±3.331 days	1 day	12 days
RIPASA score	7.721±3.23	3	13.5
Lintula score	20.486.71	10	32

**Figure 3:**
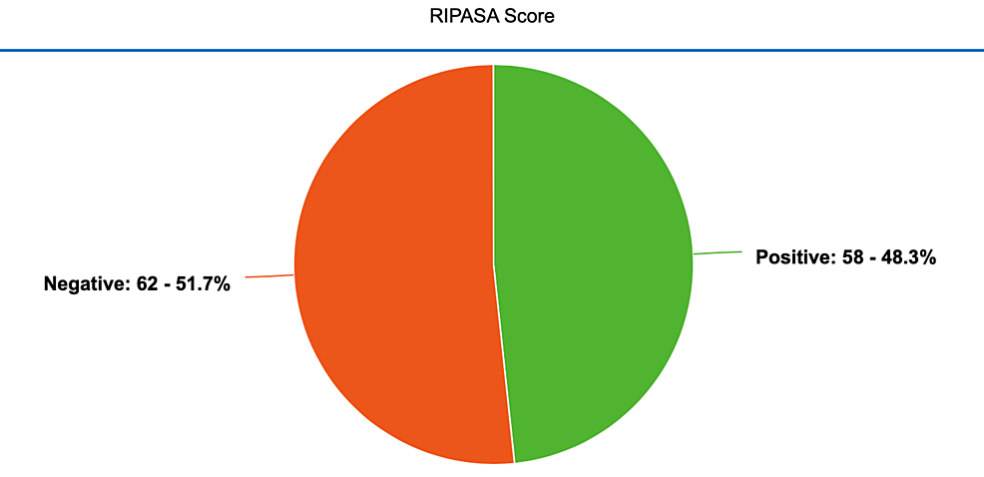
Frequency distribution of the diagnosis of acute appendicitis by RIPASA

Patients' mean RIPASA score was 7.721±3.23, having the lowest and highest values of 3 and 13.5, respectively. The RIPASA system detected acute appendicitis in 58 (48.33%) patients in this study (Figure 3).

**Figure 4:**
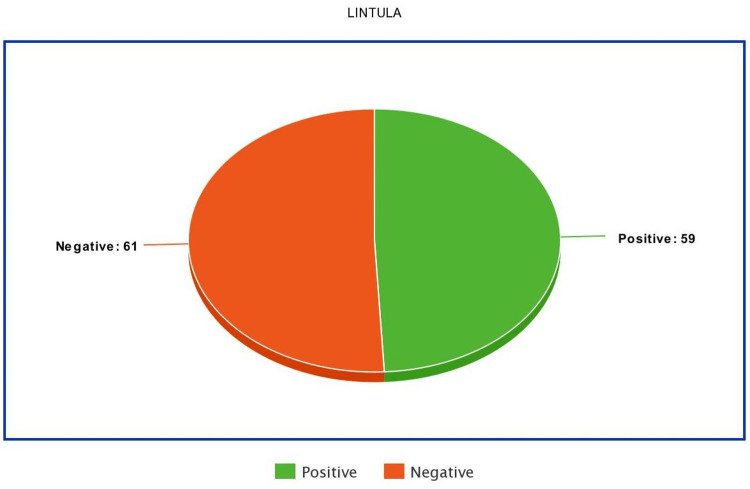
Frequency distribution of the diagnosis of acute appendicitis by Lintula

Patients' mean Lintula score was 20.486.71, with 10 as the lowest and 32 as the maximum. In our study, acute appendicitis was diagnosed by the Lintula score in 59 patients (49.2%) (Figure 4).

**Figure 5:**
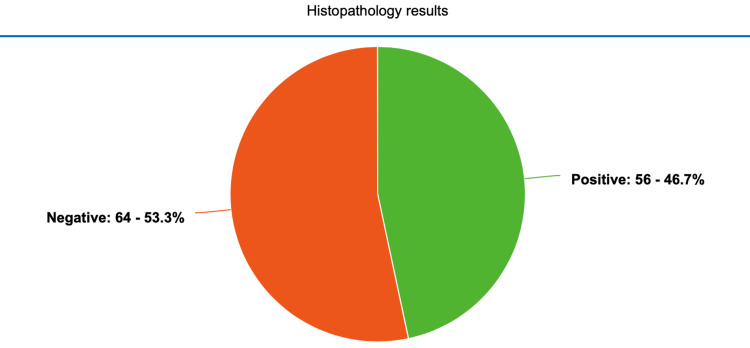
Frequency distribution by histopathology

Acute appendicitis was diagnosed by histopathology showing the presence of inflammation of the appendix, multiple micro-abscesses of the appendiceal wall and necrosis of layers in the organ. Out of 120, 56 (46.67%) patients were found to have acute appendicitis on the histology specimen (Figure 5).

Taking histopathology as the gold standard, the study found that the sensitivity, specificity, and diagnostic accuracy of the RIPASA score to detect acute appendicitis were 92.86%, 90.63%, and 91.67%, respectively. The PPV of the RIPASA score was 89.66% and NPV was 93.55%. The Lintula score had a sensitivity, specificity, and diagnostic accuracy of 80.36%, 78.13%, and 79.17%, respectively, for detecting acute appendicitis. The PPV of the Lintula score was 76.27% and NPV was 81.97% (Table 4).

**Table 4:** Validity of both scores for the detection of acute appendicitis taking histopathology as the gold standard

	RIPASA	Lintula
Sensitivity	92.86%	80.36%
Specificity	90.63%	78.13%
Positive predictive value	89.66%	76.27%
Negative predictive value	93.55%	81.97%
Diagnostic accuracy	91.67%	79.17%

**Figure 6:**
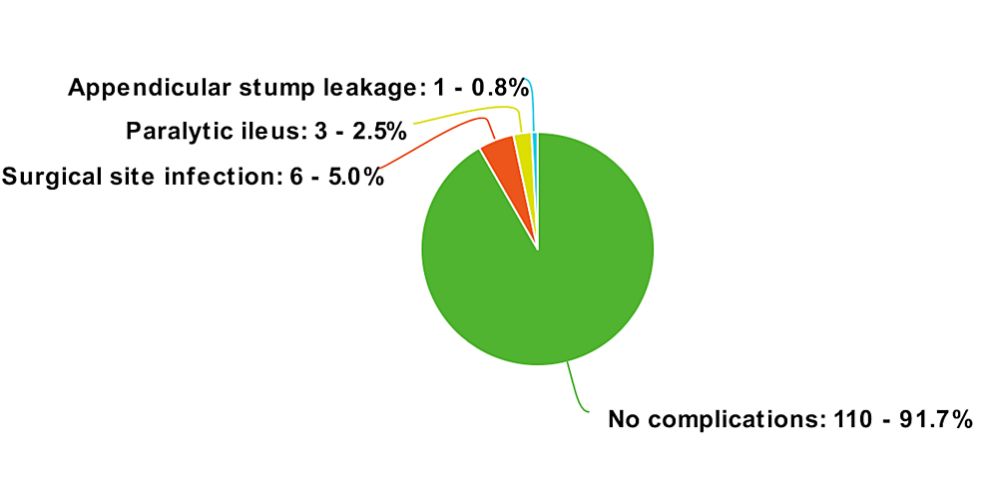
Postoperative complications

Postoperative complications included surgical site infections in 5% (n=6), paralytic ileus in 2.5% (n=3) and appendicular stump leakage in 0.8% (n=1). Surgical site infections were managed with antibiotics as per culture sensitivity and wound care while paralytic ileus was managed conservatively. One patient underwent re-exploration secondary to the appendicular stump leakage (Figure 6).

## Discussion

A common cause of severe abdominal discomfort that necessitates a rapid surgical intervention is acute appendicular inflammation, with a lifetime frequency of 1 in 7. Due to the presence of atypical presentation and symptoms, achieving a precise and early diagnosis remains a major difficulty. Various scoring systems have been established around the world to help clinicians make an accurate diagnosis and reliably rule out acute appendicitis [11]. The sensitivity, specificity, and diagnostic accuracy of the RIPASA system to detect acute appendicitis as investigated in this research were 92.86%, 90.63% and 91.67%, respectively, when using surgical findings as the gold standard. Similarly, using surgical findings as the gold standard, the Lintula score's specificity, sensitivity, and diagnostic accuracy to detect acute appendicitis were 78.13%, 80.36% and 79.17%, respectively. Both RIPASA and Lintula scores were found to be accurate in detecting acute appendicitis in this study, although the RIPASA score outperformed the Lintula scoring system.

Chong et al. reported that a score of RIPASA >7.5 accurately diagnosed 98% of histologically proven cases of acute appendicitis, compared to 68.3% cases having a score of >7 found using the Alvarado system [12]. The RIPASA score had a negative appendectomy rate of 13.7%, a sensitivity of 97.7%, and a specificity of 77.4%, according to Khadda et al., more than most of the other studies [13].

According to an Irish study by Malik et al., RIPASA score had a negative appendectomy rate of 15.94%, negative predictive value of 72.86%, positive predictive value of 84.06% and accuracy of 80% [14]. According to Pasumarthi and Madhu, RIPASA scoring system's sensitivity, specificity, PPV and NPV were 75%, 65%, 91.14% and 35.14%, respectively, while the diagnostic accuracy was 73.28 [15]. Reyes-García et al. discovered that RIPASA had a sensitivity of 91.2% and a specificity of 84.6% [16]. Díaz-Barrientos et al., on the other hand, discovered that RIPASA had a sensitivity of 93.3% but a specificity of 8.3% for detecting acute appendicitis [17]. In accordance with a different research, the RIPASA score had a poor sensitivity and specificity of 75% and 65%, respectively, for predicting acute appendicitis [15]. Elhosseiny et al. found that the RIPASA score to diagnose acute appendicitis showed 100% sensitivity, 88% accuracy, 100% negative predictive value and 4.1% negative appendectomy rate [18]. When it came to the Lintula score, Konan et al. found that the Lintula score was highly sensitive and specific to diagnose acute appendicitis in individuals over 65 years of age [19].

With respect to a prospective research conducted at the Kenyatta National Hospital, Nairobi, Ojuka and Sangoro discovered that the Lintula and Alvarado scores had very identical receiver operating characteristic (ROC) curves (0.6824 and 0.6966, respectively) [11]. They discovered that the Lintula and Alvarado scoring systems had a sensitivity of 60.8% and 84.3%, respectively, and specificity of 60% and 35%. Lintula's PPV was 79.5%, while Alvarado's was 76.8%.

Kırkıl et al. examined the Lintula and Alvarado scoring systems and found that the Lintula system had a sensitivity of 83.9% and the Alvarado had a sensitivity of 95.5% (p=0.007) [20]. The sensitivity of Lintula was noted to be 88.1% with a specificity of 91.7% for appendicitis prediction [21]. In another research, the Lintula score was found to be 100% sensitive and 88.4% specific for predicting appendicitis [22].

## Conclusions

Many scoring systems are used to identify patients with acute appendicitis. By far the most common used is the Alvarado score. Acute appendicitis is a clinical diagnosis and no scoring system can replace the clinical judgement. These criteria are merely meant to guide clinicians towards the suspected diagnosis. In this study, both the RIPASA and the Lintula scoring systems performed well in diagnosing acute appendicitis; however, the RIPASA scoring system outperformed the Lintula scoring system when surgical findings were used as the gold standard.
